# The effect of modern PET technology and techniques on the EANM paediatric dosage card

**DOI:** 10.1007/s00259-021-05635-2

**Published:** 2021-12-15

**Authors:** John Dickson, Uta Eberlein, Michael Lassmann

**Affiliations:** 1 Institute of Nuclear Medicine, University College London Hospitals, London, UK; 2 Department of Nuclear Medicine, University Hospital Würzburg, Würzburg, Germany

**Keywords:** PET, EANM dosage card, PET/MR systems

## Abstract

**Aim:**

Recent advancements in PET technology have brought with it significant improvements in PET performance and image quality. In particular, the extension of the axial field of view of PET systems, and the introduction of semiconductor technology into the PET detector, initially for PET/MR, and more recently available long-field-of-view PET/CT systems (≥ 25 cm) have brought a step change improvement in the sensitivity of PET scanners. Given the requirement to limit paediatric doses, this increase in sensitivity is extremely welcome for the imaging of children and young people. This is even more relevant with PET/MR, where the lack of CT exposures brings further dose reduction benefits to this population. In this short article, we give some details around the benefits around new PET technology including PET/MR and its implications on the EANM paediatric dosage card.

**Material and methods:**

Reflecting on EANM adult guidance on injected activities, and making reference to bed overlap and the concept of MBq.min bed^−1^ kg^−1^, we use published data on image quality from PET/MR systems to update the paediatric dosage card for PET/MR and extended axial field of view (≥ 25 cm) PET/CT systems. However, this communication does not cover the expansion of paediatric dosing for the half-body and total-body scanners that have recently come to market.

**Results:**

In analogy to the existing EANM dosage card, new parameters for the EANM paediatric dosage card were developed (class B, baseline value: 10.7 MBq, minimum recommended activity 10 MBq). The recommended administered activities for the systems considered in this communication range from 11 MBq [^18^F]FDG for a child with a weight of 3 kg to 149 MBq [^18^F]FDG for a paediatric patient weight of 68 kg, assuming a scan of 3 min per bed position. The mean effective dose over all ages (1 year and older) is 2.85 mSv.

**Conclusion:**

With this, recommendations for paediatric dosing are given for systems that have not been considered previously.

## Introduction

Molecular imaging plays an important role in the diagnosis of many paediatric disorders including urology, orthopaedics, oncology, endocrinology or neurology. These methods reveal physiological processes in vivo, allow early detection of disease, assist in patient management and therapeutic decisions, and provide an important tool to follow the success of therapy or to assess progression of disease [1]. The administration of radiopharmaceuticals to children exposes them to low levels of ionizing radiation. Although there is no direct evidence demonstrating adverse health effects in humans for the levels of radiation exposure associated with medical imaging, many consider it prudent to optimize the exposure to patients undergoing these studies. In addition, children are thought to be at a higher risk to ionizing radiation than adults [2, 3].

The European Association of Nuclear Medicine (EANM) has developed guidelines on paediatric activity dosing since the late 1990s leading to the publication of the first versions of the EANM paediatric dosage card in 2007 [4, 5]. Independently, the North American Consensus Guidelines (NACG) were developed for administered activities for children and adolescents in 2011 [6]. In 2014, an effort to harmonize the EANM dosage card and the NACG resulted in new published versions of each [7]. In 2016, the EANM dosage card was amended for ^68^Ga-labelled compounds, based on a publication by Machado et al. [8]. The NACG were also amended around this same time [9].

Recently, PET/MR systems have been developed and come into clinical use. It has been suggested that such systems could play a major role in paediatric nuclear medicine because, unlike PET/CT systems, they do not require the additional radiation exposure from a CT for attenuation correction and localisation [10]. Due to the longer field of view and therefore the improved sensitivity of these systems, there are three publications describing a potential reduction of the injected activity for [^18^F]FDG in a paediatric population [11–13] compared to the current version of the EANM paediatric dosage card.

Independently, for [^18^F]FDG, the EANM developed procedure guidelines for tumour imaging in adults [14]. In this guideline, techniques that considered bed overlap and scan duration for modifying activity administration were introduced. Furthermore, in the most recent version, some guidance has been given to include long field of view PET systems with “digital” detectors, which have an inherently higher sensitivity compared to older systems.

In 2021, two publications reassessed the [^18^F]FDG activity to be injected for a paediatric population for a contemporary conventional PET/CT system with an extended axial field of view of 21.8 cm [15, 16]. For this system, both studies concluded that the recommended activity values of the paediatric dosage card for this specific system could be lowered considerably and are in the same range or lower than the activities recommended by the EANM dosage card for 3D mode scanners using the [^18^F]FDG brain study entry.

As technology is changing rapidly (e.g., PET/MR, semiconductor PET technology, long field of view PET), the purpose of this work is to review what adaptions to the current version of the EANM dosage card (Version 5.7.2016) may be required to best reduce administered activity in paediatric patients for this equipment. This includes the discussion of a scan-duration adapted dosing concept for PET/MR and PET/CT which becomes increasingly common because of the variety of scan times used in clinical routine.

### Comparison of adult and paediatric guidelines for injected activity

The EANM dosage card is designed to achieve the same effective dose irrespective of the child’s size. This is achieved by using a weight-based scaling dependent on radiopharmaceutical, together with a minimum activity [4]. In adult PET, weight-based scaling is also used. However, in that case, the emphasis is more on achieving similar levels of image noise across different patient sizes. This concept was disregarded in the EANM paediatric dosage card because it can lead to excessive effective doses [17], although this approach has been questioned for PET [18]. Instead, to achieve an acceptable level of image noise in paediatric imaging, increasing the scanning duration from that used in adults can be useful.

In the EANM adult tumour PET imaging guidelines [14], in addition to linear (and non-linear) weight-based scaling of injected activity, the concept of including scan duration has been incorporated, together with differing factors depending on the applied bed overlap. The latter being suggested because of the influence of overlap on image noise in whole body PET imaging [19]. Given the history of the EANM dosage card project, and the generality of its approach to cover all nuclear medicine and PET investigations, the dosage card did not explicitly suggest a scan time and bed overlap. Instead, it assumed the same scan duration as for adults. Based on the literature at the time the dosage card was written, for PET imaging, a scanning duration of 3 min per bed position and manufacturer recommended bed overlaps were advised. While scanning times are relatively standard at around 3 min per bed position at many PET centres, any adjustments that reduce or increase scan duration should come with corresponding adjustments to injected activity. While the dosage card is applicable for standard bed overlaps, given the range of bed overlaps provided by the manufacturers (~ 20% to ~ 50%), further optimisation in this area is still possible, potentially using the recommendations of the EANM adult guidelines as a starting point. For sites that use continuous bed motion PET acquisitions [20], manufacturer recommended table speeds and/or noise equivalent count (NEC)-based conversions between time per bed position and overlap to table speed should be used [21].

### The potential effect of PET technology developments on paediatric injected activity

In recent years, there has been a significant shift in PET hardware technology, from traditional PET/CT systems to the introduction of PET/MR, “digital” semiconductor readout of light from the scintillation crystals, and longer (≥ 25 cm) axial field of view detector rings (Table 1). While the current EANM dosage card is still relevant and applicable with these shifts in technology, the improved sensitivity that comes with these developments allows the potential reduction of injected activities.

**Table 1:** A selection of PET/CT scanners with referenced performance characteristics. (*BGO* bismuth germanate, *LSO* lutetium-oxyorthosilicate, *LBS* lutetium based scintillator, *PMT* photomultiplier tube, *APD* avalanche photodiode, *SiPM* silicon photomultiplier, *ToF* time of flight

*System*	*Axial FoV (cm)*	*Sensitivity (cps/kBq)*	*Crystal*	*Readout*	*ToF resolution (ps)*	*Reference*
***Traditional PET/CT systems***						
*GE Discovery STE*	15.7	8.9*	BGO	PMT	-	[22, 23]
*Philips Gemini*	18	6.6	LYSO	PMT	585	[24, 25]
*GE Discovery 690*	15.7	7.6	LBS	PMT	544	[26]
*Siemens mCT*	21.8	9.7	LSO	PMT	530	[27]
*Philips Ingenuity*	18	7.4	LYSO	PMT	502	[28]
***PET/MR systems***						
*Siemens Biograph mMR*	25.8	15	LSO	APD	-	[29]
*GE Signa*	25	23.3	LBS	SiPM	< 400	[30, 31]
*United Imaging uPMR790*	32	15.9	LYSO	SiPM	535	[32]
***Contemporary PET/CT systems with semiconductor readout***						
*Philips Vereos*	16.4	5.2	LYSO	SiPM	310	[33]
*GE Discovery MI 4R*	20	13.5	LBS	SiPM	380	[34]
*Siemens Vision 450*	19.7	9.1	LSO	SiPM	215	[35]
***Long field of view PET/CT systems with semiconductor readout***						
*Siemens Vision 600*	25.6	16	LSO	SiPM	215	[36]
*GE Discovery MI 5R*	25	20.7	LBS	SiPM	380	[37]

*Performance in 3D acquisition mode.

While 15–18 cm axial field of view systems were traditionally standard, the extension of the PET detector ring beyond 20 cm and now 25 cm on PET/MR and PET/CT systems has improved performance. Extending the axial field of view allows the scanner to capture more lines of response from the positron emissions, which in turn improves the systems sensitivity almost by the square of the length. As shown in Table 1, whereas sensitivity measurements of around 6–9 kcps/kBq have been common in traditional systems with axial field of views less than 20 cm, the sensitivity of contemporary long field of view (≥ 25 cm) PET/CT and PET/MR scanners is 2–3 times this figure at 16–23 cps/kBq. Also of note is the relatively similar sensitivities of these long field of view PET/MR and PET/CT systems.

The transition from traditional photomultiplier systems to semiconductor SiPMs detectors has seen further gains in PET performance. New scintillation detectors using SiPMs found in both PET/CT and PET/MR systems have a slightly higher sensitivity but, more significantly, are capable of timing resolutions close to 200 ps. The latter translates into significant improvements to time-of-flight PET performance [38] and associated signal to noise benefits [39].

### Consequences for the paediatric injected activity for PET/MR and long field of view scanners

For the three PET/MR scanner systems (Siemens Biograph mMR, GE Signa, UI uPMR790) and long-field-of-view scanners (≥ 25 cm) with semiconductor readout (Siemens Vision 600, GE Discovery MI 5R) currently available, the sensitivities are higher than those for conventional systems (see Table 1).

At the time of writing, there were three publications, describing a potential reduction of the injected activity for [^18^F]FDG PET/MR in a paediatric population [11–13]. Overall, in these three papers, 52 patients were scanned with two different PET/MR scanner types. Gatidis et al. [11] (24 patients) scanned at 4 min per bed position with an activity of 3.1 MBq/kg. They found that an administered activity of 1.5 MBq/kg was sufficient for adequate results. This value corresponds to 6 MBq.min bed^−1^ kg^−1^ (4 min × 1.5 MBq/kg). Zucchetta et al. [12] (17 patients) applied 5 min per bed position with an administered activity of 3 MBq/kg body weight. They also found that an administered activity of 1.5 MBq/kg is adequate for sufficient image quality (7.5 MBq.min bed^−1^ kg^−1^). Both groups used a SIEMENS Biograph mMR. Schmall et al. [13] (11 patients) injected 3.7 MBq/kg with a bed overlap of 33%, using a GE Signa scanner. They found adequate image quality for an administered activity of 2.5 MBq/kg, however for a scan duration of 3 min per bed position (7.5 MBq.min bed^−1^ kg^−1^). In each study, image quality was assessed based on its visual impression. In addition, the study of Zucchetta et al. [12] demonstrated by using several image quality metrics the potential of reducing the activity in clinical PET applications.

It can be concluded that an injected activity between 6 and 7.5 MBq.min bed^−1^ kg^−1^ is sufficient to provide adequate image quality for a standard bed overlap. As a result, we recommend using the equivalent of 2.5 MBq/kg for a 3 min/bed position for both PET/MR scanners, provided the bed overlap is similar to the values reported [13]. For different scanner types, bed overlaps, and scan duration per bed, the activity values should be adjusted accordingly. For example, an increase of the scan time to 5 min per bed would result in an activity that could be reduced to 60% of the calculated value. The activities are calculated for commonly used PET/MR systems and currently available long-field-of-view scanners (≥ 25 cm) with semiconductor readout. Systems with a long-field-of-view of more than 32 cm are not within the scope of this recommendation, although they may be addressed at a later time.

The dosage recommendations should be taken in context of “good practice” of nuclear medicine and do not substitute for national and international legal or regulatory provisions. Lower activities can be administered if the PET systems used suits the needs of the clinic with respect to their specific equipment, clinical preference, and the particular needs of their patients.

In order to be consistent with the previous versions of the EANM dosage card and the methodology described [4, 5, 7, 8], the following reduced values for [^18^F]FDG PET/MR and long-field-of-view PET/CT are proposed for a scan duration of 3 min per bed position:

#### Class B, baseline value: 10.7 MBq, minimum recommended activity: 10 MBq

The best match of the formalism provided by the dosage card to a 2.5 MBq/kg dosing regimen resulted in a mean activity reduction of 41.2% for all weights. Consequently, the baseline value of 25.6 MBq provided by the dosage card for [^18^F]FDG torso was reduced by this factor.

Table 2 shows the recommended activities and effective doses, taken from ICRP128 [40], for PET/MR (and long field of view PET/CT scanners) in comparison to an activity of 2.5 MBq/kg. In order to be consistent with the previous values and due to the lack of data for effective doses according to the weighting factors of ICRP103 [41], the tissue weighting factors of ICRP60 were applied [42].

**Table 2:** Recommended administered activities (dosage card vs. weight-based) and effective dose values taken from ICRP128 [40], for a 3 min per bed position acquisition. For comparison, the values of the EANM dosage card [7] and the NA consensus guideline are provided [43]

Age	Newborn	1 year	5 years	10 years	15 years	Adult
Weight (kg)	3	10	19	32	57	68
Administered activity (MBq)	11	29	50*	78	124*	149
Effective dose (mSv)	NA	2.75	2.80	2.88	2.98	2.84
**For comparison:**						
2.5 MBq/kg administered activity (MBq)	7.5	25	48	80	138	170
EANM dosage card 3D mode [5]/[^18^F]FDG PET of the brain [7]* (MBq)	14	38	65	102	163	200
EANM dosage card 2D mode [5]/[^18^F]FDG of the torso [7]* (MBq)	26	70	120	189	302	363
North American consensus guidelines (5.2 MBq/kg) [43] (MBq)	26	52	98	166	295	352

*Interpolated values.

## Summary

Developments in imaging technology have brought welcome performance enhancements to our field. Recent advances in PET technology such as the expansion of the axial field of view from 15 to 25 cm and faster semiconductor processing of light emanating from the scintillation crystal, first for PET/MR and then for PET/CT, have brought with it significant gains in scanner performance and sensitivity. Something that is very relevant for helping to reduce radiation exposure in paediatric imaging.

This communication has highlighted the scale of some of these sensitivity gains, and using available publications has made a recommendation of the adaption of the current dosage card to provide new factors for these new long field of view semiconductor readout PET/MR and PET/CT systems. Also touched on in this paper is the consideration that should be made to the duration of the scan and the bed overlap being used, bringing alignment with the adult tumour guidelines. The duration of the scan being something incredibly relevant for PET/MR scanning where duration of the MR component may be significantly longer than the PET component.

What this communication does not cover is (i) a reassessment of the EANM dosage card for conventional scanners and (ii) the expansion of paediatric dosing for the half-body and total-body scanners that have recently come to market. Nor does it move to a simple dose per kilogram model which would be inconsistent with the rest of the dosage card. However, it is hoped that future endeavours will reassess the recommended activity values for contemporary conventional systems and address longer axial field of view and more sensitive scanners using the methodology provided in the adult FDG tumour guideline [14], while the investigation of a move to simpler weight-based models could also be addressed.
